# Bright on Cancer, Its Classification and Remedies

**Published:** 1871-12

**Authors:** 


### Books Reviewed.

*Bright on Cancers, its Classification and Remedies.* Published by S. W. Butler, M. D., Philadelphia, Pa., 1871.

The aim of the author, he says, "has been to place in the hands of the profession a work that will save much labor in collecting facts, and at the same time give a classification that will remove the difficulty of having so many names for the same form of cancer." His idea of classification seems fully expresed in the phrase, "cancer is a morbid growth containing the cancer cell." His therapeutics is briefly announced "to remove cancer you must remove every cancer cell," and his treatment is expressed in numbers as follows:

"With this explanation I shall proceed to give the remedies and their compounds. That they may be the more easily recollected, I will number them, as far as the general remedies are used, so that when a number is referred to, the exact remedy used may be known, and applied in each case and class.

No. 1. Solid extract of podophyllum (the root), one part; pure choloride zinc, three parts; starch, ene-fourth part; red saunders, one-fourth part; water sufficient to form a thick paste. The object of the starch is to give tenacity to the paste, and the red saunders to give porosity, that the plaster may not run, and at the same time give porosity enough to let the full effect of the active articles pass to the sore, where they are rapidly absorbed. This preparation will keep any length of time in a glass or porcelain cup.

No. 2. This is simply a saturated solution of chloride of zinc. It must be chemically pure, kept in a glass-stoppered bottle, and when applied, a glass brush should be used.

No. 3· This is a paste like No 1, with the exception of using carbolic acid instead of water.

No. 4. This is an arrow made of pure chloride of zinc. Take enough starch to absorb the moisture of the chloride, work together on a pillplate with a wooden spatula, just enough starch being added to make a stiff paste. Roll the paste into thin cakes, and cut the arrows to a point; then roll gently, and dry on a plate at 212° Fahrenheit. These arrows, properly prepared, and put into a glass-stoppered bottle, will keep any length of time.

These are the potent remedies generally used in in open cancer; all others are only auxiliries, but, in their place, of great importance. We shall give them when we give cases, etc. We shall only give a few cases of each class, deeming that sufficient to guide any one who wishes to try the remedies. We have classified cancer, and shall not repeat the classification.

Case I.—Mr. Webb, August, 1864, called on me with a sore on his cheek, which had existed for several months. Upon examination, I found it filled with cancer cells. This was an epithelioma. The sore was raw, and the edges everted. I made three applications of No. 1 plaster, spread on a piece of cotton cloth fully large enough to cover the sore and margin; confined it with adhesive straps. It was removed once in twenty-four hours. After the third application the surfface of the sore was white and hard. I then ordered a poultice of light bread and water to be applied, and to be renewed every six hours. In six days the cancer fell out, leaving the edges and bottom smooth. The lump that came out was one inch in diameter, and more than half an inch thick in the centre. By dressing it with simple cerate it healed in due time, and left a smooth scar. It remains well."

The author talks very like an itinerant cancer doctor; seems to think he has received a new revelation, says: " I am persuaded that when fifty years ago I cut out cancers, I did not cure ten per cent. of them, while now, one thing I know, I have not for the last twenty years lost ten per cent. of my patients."

The publisher makes a note on condurango, repeating the old story (Bliss, Colfax & Co.'s story) and says: " If all is true that is claimed for it the great dissideratum pointed to by the author of this book—the discovery of an agent that will reach the specific virus of cancers and neutralize it—is already met.

The work contains valuable resume of the history, nature and various plans adopted for the treatment of cancer, but his favorite plan of treatment requires confirmation, and his positive declarations of cure would sound better if applied to some other disease more often know to terminate favorably.

